# Glutamine Metabolism in Both the Oxidative and Reductive Directions Is Triggered in Shrimp Immune Cells (Hemocytes) at the WSSV Genome Replication Stage to Benefit Virus Replication

**DOI:** 10.3389/fimmu.2019.02102

**Published:** 2019-09-04

**Authors:** Shu-Ting He, Der-Yen Lee, Cheng-Yi Tung, Chun-Yuan Li, Han-Ching Wang

**Affiliations:** ^1^Department of Biotechnology and Bioindustry Sciences, College of Biosciences and Biotechnology, National Cheng Kung University, Tainan, Taiwan; ^2^Graduate Institute of Integrated Medicine, China Medical University, Taichung, Taiwan; ^3^International Center for the Scientific Development of Shrimp Aquaculture, National Cheng Kung University, Tainan, Taiwan

**Keywords:** white spot syndrome virus, IDHs, oxidative glutaminolysis, reductive carboxylation, hemocytes

## Abstract

White spot syndrome virus (WSSV) is the causative agent of a shrimp disease that has caused huge global economic losses. Although its pathogenesis remains poorly understood, it has been reported that in the shrimp immune cells (hemocytes) targeted by WSSV, the virus triggers both the Warburg effect and glutamine metabolism at the WSSV genome replication stage (12 h post infection). Glutamine metabolism follows two pathways: an oxidative pathway mediated by α-KGDH (α-ketoglutarate dehydrogenase) and an alternative reductive pathway mediated by IDH1 and IDH2 (isocitrate dehydrogenase 1 and 2). Here we used isotopically labeled glutamine ([U-^13^C]glutamine and [1-^13^C]glutamine) as metabolic tracers to show that, at the replication stage, both the oxidative and reductive glutamine metabolic pathways were activated. We further show that the mRNA expression levels of α-KGDH and IDH1 were increased in WSSV-infected shrimps and that silencing of α-KGDH, IDH1, and IDH2 with their respective dsRNAs led to a decrease in WSSV gene expression and WSSV replication. Taken together, our findings provide new evidence for WSSV-induced metabolic reprogramming in hemocytes and demonstrate its importance in virus replication.

## Introduction

Although in many kinds of tumors and cancers, metabolic reprogramming has been found to be crucial for the cell's aberrant proliferation and potentially unlimited self-renewal (1, 2), immune cells are also able to control pathogens by triggering particular metabolic pathways to aid immune responses, such as increasing amino acid catabolism (3). However, there is also increasing evidence that some vertebrate viruses, especially oncogenic viruses can utilize the reprogramming of host metabolism to complete their replication cycle (4, 5). Quite recently, the white spot syndrome virus (WSSV), a large dsDNA virus that is the causative agent of a devastating viral shrimp disease, became the first invertebrate virus that was shown to induce host metabolic reprogramming in shrimp immune cells (hemocytes). These changes include the Warburg effect (aerobic glycolysis), amino acid catabolism (i.e., glutaminolysis [glutamate-driven anaplerosis]), lipid metabolism, activation of the pentose phosphate pathway, nucleotide biosynthesis, and amino acid biosynthesis (6–9). The effects are most noticeable at the genome replication stage (12 h post infection), and like the metabolic reprogramming that is seen in cancer cells and cells infected by some vertebrate viruses (10–13), the WSSV-induced metabolic changes benefit the virus by meeting the both its energy requirements and its biosynthetic needs.

When aerobic glycolysis is activated, most of the carbon atoms from the glucose that has been taken up become diverted into lactate production instead of being routed into the mitochondrial TCA (tricarboxylic acid) cycle (2, 14). Thus, in order to allow the TCA cycle to continue to produce energy and biosynthetic products during aerobic glycolysis, glutamine, an amino acid that is abundant in the circulation system, is used as an alternative carbon source in a process known as glutamine anaplerosis (11, 15, 16). In normal cells, after glutamine is converted (via glutamate) into α-KG, it is subsequently metabolized to succinate by α-ketoglutarate dehydrogenase (α-KGDH) through oxidative glutaminolysis. However, cells undergoing the Warburg effect are also able to convert α-KG into isocitrate either by cytoplasmic IDH1 or by mitochondrial IDH2 through the reductive glutamine metabolic pathway (17, 18). Although mammalian studies have shown that glutamine metabolism can have both immune-related benefits as well as pathogenesis-related effects (3–5), the pathway primarily seems to favor virus replication in shrimp infected by WSSV (8).

We have recently shown that glutamine anaplerosis also occurs in WSSV-infected shrimp: we found that WSSV increases the expression of GDH, an enzyme which converts glutamate into α-KG (8), and also that direct *in vivo* replenishment of α-KG rescued WSSV replication after the down-regulation of GDH by dsRNA-mediated gene silencing. These results all suggest that, in conjunction with the WSSV-induced Warburg effect, WSSV-infected cells may activate glutamine metabolism to fuel the TCA cycle. However, it was not known whether WSSV triggered glutamine metabolism in both the oxidative and reductive directions. In the present study, we therefore look more closely at the glutamine metabolism induced by WSSV. To do this, we use LC-ESI-MS and isotopically labeled glutamine (uniformly-^13^C [U-^13^C] glutamine and [1-^13^C] glutamine) as metabolic tracers. We also provide additional evidence of the importance of the reductive carboxylation glutaminolysis for virus replication.

## Materials and Methods

### Experimental Animals and WSSV Inoculum

The shrimp (*Litopenaeus vannamei*) of around 3 g body weight used in the study were obtained from the International Center for the Scientific Development of Shrimp Aquaculture, National Cheng Kung University (NCKU), and the Department of Aquaculture, National Pingtung University of Science and Technology (NPUST). Before the experiments, shrimp were cultured for 1~3 days in sterilized seawater (30 ppt at 26~27°C). The WSSV (Taiwan isolate, GenBank accession no. AF440570) stock (3.3 × 10^4^ WSSV copies/μl) was prepared from hemolymph of WSSV-infected moribund SPF (specific pathogen free) shrimp as described previously (6, 9). The viral inoculum was prepared from the stock for intramuscular injection by dilution (10^−4^) with 1x PBS (137 mM NaCl, 2.7 mM KCl, 10 mM Na_2_HPO_4_, 2 mM KH_2_PO_4_). The WSSV challenge dosage (100 μl/3 g shrimp) resulted in an ~50% cumulative mortality at 3 days post WSSV challenge. Shrimp in the control group were treated with PBS (100 μl/3 g shrimp). At 12 and 24 h post WSSV challenge, hemocyte samples and stomach tissue were collected and used for the glutamine-metabolism-related enzyme activity assays. The hemocyte samples were also used to measure the expression of host genes and viral genes, for the stable-isotope metabolic tracing experiments, and to measure the copy number of the WSSV genomic DNA as described in Su et al. (9).

### Cloning of Full-Length cDNA of *Lv*GLS1, *Lv*GLS2, *Lv*IDH1, *Lv*IDH2, and *Lv*α-KGDH

By using next generation sequencing, an in-house *L. vannamei* stomach transcriptomic database was established (data not shown) and this was used to search for the target genes. Two contigs, PVHP259804.2 and PVHP193998, were found to show high homology with Rat GLS (Accession number: M65150.1), and PVHP193998 also matched *P. vannamei* GLS (Accession number: XP_027218904.1) with 98% identity. These contig sequences were used to design primer sets to amplify the two GLS isoforms (Table 1) and these two genes were named LvGLS1(PVHP259804.2) and LvGLS2 (PVHP193998). Three other contigs, PVHP107410.1, PVHP176973.1, and PVHP203127.2, respectively showed high homology with *Penaeus vannamei* IDH1 (Accession number: XP_027219531.1), *Penaeus vannamei* IDH2 (Accession number: XP_027239404.1), and *Penaeus vannamei* α-KGDH (Accession number: XP_027220285.1). These contig sequences were also used to design primer sets to amplify the corresponding genes (Table 1).

**Table 1:** Primer sets used in the present paper.

**Gene**	**Primer**	**Primer sequence (5^**′**^-3^**′**^)^**a**^**	**Usage**
EF1α			
	EF1α-F	5′-ATGGTTGTCAACTTTGCCC-3′	Cloning
	EF1α-R	5′-TTGACCTCCTTGATCACACC-3′	Cloning
	EF1α-qF	5′-ACGTGTCCGTGAAGGATCTGAA-3′	Real-time PCR
	EF1α-qR	5′-TCCTTGGCAGGGTCGTTCTT-3′	Real-time PCR
LUCIFERASE			
	Luc-F	5′-CTGAATACAAATCACAGAATC-3′	Cloning
	Luc-R	5′-GTAAGACCTTTCGGTACTTCG-3′	Cloning
	T7-Luc-F	5′-TAATACGACTCACTATAGGGAGACTGAATACAAATCACAGAATC-3′	dsRNA synthesis
	T7-Luc-R	5′-TAATACGACTCACTATAGGGAGAGTAAGACCTTTCGGTACTTCG-3′	dsRNA synthesis
GDH			
	GDH-qF	5′-TGAGGAGAAGCGCAACAAGA-3′	Real-time PCR
	GDH-qR	5′-TGGCAGGGCTCCATGATC-3′	Real-time PCR
GLS1			
	GLS1-qF	5′-CATTGGCGACACTGACAT-3′	Real-time PCR
	GLS1-qR	5′-CTGCAGAAGGCCATTGACTA-3′	Real-time PCR
GLS2			
	GLS2-F	5′-GACCGCAAGAACCTCCTCAA-3′	Cloning
	GLS2-R	5′-GTGATGACAGAAGCCACGGA-3′	Cloning
	T7-GLS2- F	5′-TAATACGACTCACTATAGGGAGAGACCGCAAGAACCTCCTCAA-3′	dsRNA synthesis
	T7-GLS2- R	5′-TAATACGACTCACTATAGGGAGAGTGATGACAGAAGCCACGGA-3′	dsRNA synthesis
	GLS2-qF	5′-AACTACATGGGGATGGAG-3′	Real-time PCR
	GLS2-qR	5′-GATTGAAATCCAGGCAAAGCTC-3′	Real-time PCR
IDH1			
	IDH1-F	5′-GAGGATTTGCTCATGCTTC-3′	Cloning
	IDH1-R	5′-TCAGGCAGTGATCTTCTTCTGC-3′	Cloning
	T7-IDH1-F	5′-TAATACGACTCACTATAGGGAGAGAGGATTTGCTCATGCTTC-3′	dsRNA synthesis
	T7-IDH1-R	5′-TAATACGACTCACTATAGGGAGATCAGGCAGTGATCTTCTTCTGC-3′	dsRNA synthesis
	IDH1-qF	5′-GGCATGATGACCTCGGTACTG-3′	Real-time PCR
	IDH1-qR	5′-GGCAGCCTCAGACTCCAGAGT-3′	Real-time PCR
IDH2			
	IDH2-F	5′-GCAAGAACTACGATGGTG-3′	Cloning
	IDH2-R	5′-ATGCAGCCAGCCAGATCCT-3′	Cloning
	T7-IDH2-F	5′-TAATACGACTCACTATAGGGAGAGCAAGAACTACGATGGTG-3′	dsRNA synthesis
	T7-IDH2-R	5′-TAATACGACTCACTATAGGGAGAATGCAGCCAGCCAGATCCT-3′	dsRNA synthesis
	IDH2-qF	5′-CCAACCCTGTTGCTTCCATT-3′	Real-time PCR
	IDH2-qR	5′-AAGCTTGGCACGATGTTCAAG-3′	Real-time PCR
α-KGDH			
	KGDH-F	5′-ATGGGTTTGAGGCATTCTTG-3′	Cloning
	KGDH-R	5′-CCTGAGAAAGCAGCATCTCC-3′	Cloning
	T7-KGDH-F	5′-TAATACGACTCACTATAGGGAGAATGGGTTTGAGGCATTCTTG-3′	dsRNA synthesis
	T7-KGDH-R	5′-TAATACGACTCACTATAGGGAGACCTGAGAAAGCAGCATCTCC-3′	dsRNA synthesis
	KGDH-qF	5′-TCCAGCCTCGCATTTCCA-3′	Real-time PCR
	KGDH-qR	5′-GACGGCCAGCATATGAAA-3′	Real-time PCR
VP28			
	vp28-real-F	5′-AGTTGGCACCTTTGTGTGTGGTA-3′	Real-time PCR
	vp28-real-R	5′-TTTCCACCGGCGGTAGCT-3′	Real-time PCR

a *The added T7 promoter sequence is underlined*.

### Measurement of Host Genes and the WSSV Major Structural Gene VP28 by Real-Time PCR

After shrimp tissues were collected at the 12 and 24 hpi time points, total RNA was extracted and subjected to cDNA synthesis by using Superscriptase II Reverse Transcriptase (Invitrogen) and Anchor-dTv primer (Table 1). The cDNA samples were used to quantify the mRNA expression of the target genes by using real-time PCR with KAPA SYBR1 FAST Master Mix (KAPA) and the Bio-Rad detection system. The gene expression levels measured in this study were for the two GLS isoforms (GLS1 and GLS2), IDH1, IDH2, α-KGDH, the WSSV major structural gene VP28 and EF1α. The specific primer sets for each target gene are listed in Table 1. Data values were normalized to EF1α cDNA (internal control) and calculated by the 2^−Δ*CT*^ method. Statistically significant differences between groups were analyzed by Student's *t*-test as described in Tseng et al. (19).

### Measurement of the WSSV Genome Copy Number Using the IQ Real^**TM**^ WSSV Quantitative System

Shrimp hemocytes were collected from each group at the 12 and 24 hpi time points and subjected to genomic DNA extraction using a DTAB/CTAB DNA extraction kit (GeneReach Biotechnology Corp.). The viral genome copy numbers were then quantified by the real-time PCR-based IQ Real™ WSSV quantitative system (GeneReach Biotechnology Corp.). Statistical analysis was performed as described above.

### Determination of the Enzyme Activity of GLS in Shrimp Stomachs and Hemocytes During WSSV Infection

Hemocytes and stomachs from shrimp were collected at 12 and 24 h after WSSV or PBS injection (6 shrimp/pool and 4 pools/group), and the GLS activity was measured with a commercial Glutaminase Microplate Assay Kit (MyBiosource). The hemocyte and stomach samples were homogenized with 100 and 300 μl ice cold assay buffer, respectively. After centrifugation at 4°C at 13,000 g for 10 min, the cell debris was removed and the protein concentration in the supernatant was determined. The hemocyte lysates (3~52 μg) and stomach lysates (6 μg) were mixed with 200 μl substrate, and the reaction were incubated at 37°C for 1 h. The reactions were stopped by adding 300 μl Stop solution, incubating for 10 min and then centrifuging at 4°C at 8,000 g for 5 min. One hundred and thirty microliter of each supernatant was collected and mixed with a 70 μl reaction mixture containing 50 μl reaction buffer and 20 μl dye reagent. The controls were prepared as per the samples except that the protein lysates were replaced by distilled water. Standards and blanks were prepared according to the manufacturer's instructions. The absorbance of the final mixtures was measured at 450 nm. The GLS activity was calculated by the following equation: GLS activity $\text{(U/mg)}=\frac{4\times \text{T}\times \text{CStandard}\times \frac{\left(\text{ODSample}-\text{ODControl}\right)}{\left(\text{ODStandard}-\text{ODBlank}\right)}}{\text{CProtein}}$ (note: C_Standard_: reference standard [1 mg/ ml]; C_Protein_: protein concentration; OD_blank_: buffer only; T: reaction time in hours). Statistical analysis was performed as described above.

### Determination of the Enzyme Activity of α-KGDH in Shrimp Hemocytes and Stomachs During WSSV Infection

Hemocytes and stomachs from shrimp were collected at 12 and 24 h after WSSV or PBS injection (6 shrimp/pool and 4 pools/group) to measure the activity of α-KGDH. The hemocyte and stomach samples were homogenized with 100 and 200 μl ice cold KGDH assay buffer, respectively, from a commercial α-Ketoglutarate Dehydrogenase Activity Colorimetric Assay Kit (Sigma). The cell debris was removed by centrifugation at 4°C at 13,000 g for 10 min. After using a Bio-Rad protein assay to measure protein concentrations, samples with the appropriate amounts of protein (hemocyte lysate: 3.6 ~ 50 μg; stomach lysate: 10 ~ 20 μg) were collected and adjusted to a final volume of 50 μl using the KGDH assay buffer. The lysates were then mixed with 50 μl reaction mixture containing 46 μl KGDH Assay Buffer, 2 μl KGDH Developer, and 2 μl KGDH Substrate. The final mixtures were then incubated at 37°C and protected from light. The NADH standards supplied with the kit were prepared in the same way as the shrimp tissue samples. α-KGDH activity was measured at A450 every 3–5 min until the value of the most active sample was greater than the value for the NADH standard with the highest concentration (12.5 nmole). The NADH standard curve at this time point was then used to convert the difference in absorbance at the initial (T_initial_) and final (T_final_) time points to the amount of NADH (B). The activity was calculated by the following equation: enzyme activity (mU/mg) = B/([T_final_ – T_initial_] × [total amount of protein in the reaction]). Statistical analysis was performed as described above.

### Determination of the Enzyme Activity of IDH in Shrimp Stomachs and Hemocytes During WSSV Infection

Hemocytes and stomachs from shrimp were collected at 12 and 24 h after WSSV or PBS injection (6 shrimp/pool and 4 pools/group) to measure the activity of IDH. The hemocyte and stomach samples were homogenized with 100 and 200 μl ice cold IDH assay buffer, respectively, from a commercial Isocitrate Dehydrogenase Activity Assay Kit (Sigma). The cell debris was removed by centrifugation at 4°C at 13,000 g for 10 min. After using a Bio-Rad protein assay to measure protein concentrations, samples with the appropriate amounts of protein (hemocyte lysate: 15 ~ 30 μg; stomach lysate: 10 μg) were collected and adjusted to a final volume of 50 μl using IDH assay buffer. The lysates were then mixed with 50 μl reaction mixture containing 38 μl IDH Assay Buffer, 8 μl Developer 2 μl IDH Substrate, and 2 μl NADP^+^. The final mixtures were incubated at 37°C and protected from light. The NADH standards supplied with the kit were prepared in the same way as the tissue samples. IDH activity was measured at A450 every 3–5 min until the value of the most active sample was greater than the value of the standard with the highest concentration (10 nmole). The difference in absorbance at the initial (T_initial_) and final (T_final_) time points was converted to the NADPH amount (B) using the NADH standard curve at the final time point. The enzyme activity was calculated by the following equation: IDH activity (mU/mg) = B/([T_final_ – T_initial_] × [total amount of protein in the reaction]). Statistical analysis was performed as described above.

### *In vitro* Synthesis of *Lv*GLS2, *Lv*IDH1, *Lv*IDH2, and *Lv*α-KGDH dsRNAs

dsRNAs were prepared as described in our previous study (9, 20). In short, the partial sequences (300~600 bp) of each host gene and the non-specific Luciferase control were amplified by PCR with the following respective primer sets: GLS2-F/GLS2-R, IDH1-F/IDH1-R, IDH2-F/IDH2-R, α-KGDH-F/α-KGDH-R, and Luc-F/Luc-R (Table 1). After obtaining the corresponding PCR amplicons, T7 promoter sequence was added to the 5′ and 3′ ends of each amplicon by a second PCR with the primer sets: T7-GLS2-F/GLS2-R, GLS2-F/ T7-GLS2-R, T7-IDH1-F/IDH1-R, IDH1-F/T7-IDH1-R, T7-IDH2-F/IDH2-R, IDH2-F/T7-IDH2-R, T7-α-KGDH-F/α-KGDH-R, α-KGDH-F/T7-α-KGDH-R, T7-Luc-F/Luc-R, and Luc-F/T7-Luc-R (Table 1). The ssRNAs were then synthesized by the T7 RiboMAX Express large-scale RNA production system (Promega) and the corresponding ssRNA pairs were mixed together to form the dsRNAs. After the dsRNAs were purified by phenol/chloroform extraction, quantified by UV spectrophotometer and checked by agarose gel electrophoresis, the final dsRNA products were stored at −80°C before use.

### *In vivo* Gene Silencing of *Lv*IDH1, *Lv*IDH2, *Lv*α-KGDH Mediated by dsRNA Interference

Shrimp (~3 g body weight) were injected with the *Lv*IDH1, *Lv*IDH2, *Lv*α-KGDH dsRNAs at a concentration of 1 μg /g shrimp. Shrimp treated with Luc dsRNA were used as the control. At 72 h (3 days) post the dsRNA injection, shrimp hemocyte samples were collected from each group and real-time PCR was used to confirm that the *Lv*IDH1, *Lv*IDH2, and *Lv*α-KGDH genes had been specifically silenced by the respective dsRNA. At the same time, the remaining shrimp in each group were challenged by injection with WSSV inoculum or PBS. At 24 h post injection, shrimp hemocyte samples were collected (3 shrimp in each sample, 4 samples for each group) and used to measure the expression of host genes, viral genes and the WSSV genome copy number. Statistical analysis was performed as described above.

### Using Stable Isotope-Labeled Glutamine Tracer and Liquid Chromatography Electrospray Ionization Mass Spectrometry (LC-ESI-MS) to Monitor Metabolites in the Hemocytes of WSSV-Infected Shrimp

**Figure 1:**
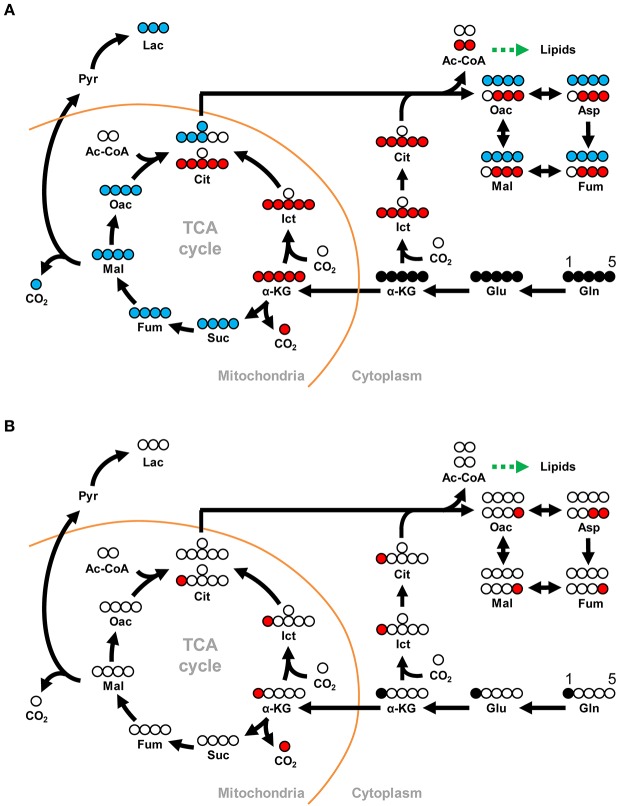
Schematic showing how the metabolites derived from **(A)** [U-^13^C]glutamine can be traced through the oxidative and reductive glutamine pathways and **(B)** [1-^13^C]glutamine can be traced through the reductive glutamine pathway. In the Gln-Glu-a-KG pathway, white circles represent ^12^Carbon, while solid black circles represent ^13^Carbon. Blue circles represent ^13^C in the oxidative glutamine metabolism pathway of the TCA cycle and the citrate shuttle, while red circles represent ^13^C in the reductive carboxylation pathways in either mitochondria or cytoplasm. The green arrow indicates the shift toward lipid metabolism. For [U-^13^C]glutamine in **(A)**, abbreviations are as follows: for the Gln-Glu-α-KG pathway: Gln, M5 glutamine; Glu, M5 glutamate; α-KG, M5 α-ketoglutarate. For oxidative glutamine metabolism: Suc, M4 succinate; Fum, M4 fumarate; Mal, M4 malate; Oac, M4 oxaloacetate; Cit, M4 citrate; Lac, M3 lactate. For reductive carboxylation: Ict, M5 isocitrate; Cit, M5 citrate; Oac, M3 oxaloacetate; Asp, M3 aspartate; Fum, M3 fumarate; Mal, M3 malate. Similar abbreviations apply for [1-^13^C]glutamine (M1 glutamine) in **(B)**.

Stable isotope-labeled glutamine can be used together with LC-ESI-MS to identify and quantify metabolites in the oxidative and reductive glutamine metabolic pathways that have incorporated the labeled glutamine carbon atoms. In this study, we used both [U-^13^C]glutamine (M5 gln) and [1-^13^C]glutamine (M1 gln) to trace the metabolites of interest as shown in Figure 1. As no shrimp cell line or alternative cell line is presently available, we established an *in vivo*, ^13^C-labeled shrimp hemocyte metabolic analysis platform *ab initio*. Based on preliminary tests (data not shown), our experimental protocol was to treat WSSV-challenged shrimp with stable isotope-labeled glutamine by haemocoel injection at 12 and 24 hpi after challenge. At 10 and 30 min after injection of the M5 or M1 gln, pooled hemocyte samples (4 pools; 3 shrimp in each pool) were collected and analyzed as described below.

Stable isotope tracer solutions were prepared by dissolving [U-^13^C]glutamine (M5 gln; Cambridge Isotope Laboratories, Inc) or [1-^13^C]glutamine (M1 gln; Cambridge Isotope Laboratories, Inc) in PBS. At 12 or 24 h after the experimental shrimp had been injected with WSSV or PBS, one of the stable isotope tracer solutions ([U-^13^C]glutamine: 400 μg /g shrimp; [1-^13^C]glutamine: 800 μg /g shrimp) was injected into the shrimp's abdominal hemal sinus. At 10 and 30 min after treatment with the tracer, 4 pooled hemocyte samples (3 shrimp in each pool) were collected from each group using a cold anticoagulant (1x PBS, 10 mM EDTA, pH8.0). After immediately centrifuging at 1,000x g for 10 min by using a swinging bucket, the pellet of shrimp hemocytes from each sample was collected, washed with ice-cold 1x PBS and the hemocytes were lysed with sterilized ddH_2_O on ice. Next, 100% MeOH was added to the cell lysate at a ratio of 1:3 to quench the metabolic reactions, and the samples were kept at −80°C for 10 min. The samples were then centrifuged at 4°C at 18,000 g for 10 min, and 75% MeOH was added to the cell lysate for secondary extraction. The supernatants, which now contained the metabolites, were then collected, lyophilized, and stored at −80°C before being analyzed by LC-ESI-MS as described previously (9).

Briefly, for LC-ESI-MS analysis the samples were dissolved in 35 μl of ddH_2_O and 5 μl of reaction buffer (0.3 M aniline [Sigma-Aldrich, USA] in 60 mM HCl), and 5 μl of N-(3-dimethylaminopropyl)-N'-ethylcarbodiimide hydrochloride (EDC; Sigma-Aldrich, USA) was added. After incubating the mixture at 25°C for 2 h, 5 μl of 10% ammonium hydroxide was added to stop the reaction. The derivatives were analyzed on an LC-ESI-MS system comprising an ultra-performance liquid chromatography (UPLC) system (Ultimate 3000 RSLC, Dionex) and a quadrupole time-of-flight (QTOF) mass spectrometer with an electrospray ionization (ESI) source (maXis HUR-QToF system, Bruker Daltonics). Reversed-phase liquid chromatography (RPLC) on a BEH C18 column (2.1 × 100 mm, Waters) was used. The elution started from 99% mobile phase A (0.1% formic acid in ddH_2_O) and 1% mobile phase B (0.1% formic acid in ACN), held at 1% B for 0.5 min, raised to 60% B in 6 min, further raised to 90% B in 0.5 min, held at 90% B for 1.5 min, and then lowered to 1% B in 0.5 min. The column was then equilibrated by pumping 1% B for 4 min. The flow rate was set at 0.3 ml/min with an injection volume of 10 μl. LC-ESI-MS chromatograms were acquired under a capillary voltage of either 4,500 or 3,500 V in negative ion mode, a dry temperature of 190°C, a dry gas flow maintained at 8 l/min, nebulizer gas at 1.4 bar, and an acquisition range of m/z 100–1,000.

Data were acquired by HyStar and micrOTOF control software (Bruker Daltonics) and processed by DataAnalysis and TargetAnalysis software (Bruker Daltonics) to generate the signals corresponding to the integrated areas for each extracted ion chromatogram.

To monitor the change in the quantity of the ^13^C labeled metabolites, fold changes in the WSSV group were calculated relative to the corresponding PBS group (WSSV/PBS group). All the signal counts were normalized by the sample's weight (mg). Statistically significant differences between WSSV and PBS groups were analyzed by Student's *t*-test as described above.

### Effect of the Inhibitors Salirasib (S35), Torin1, and LY294002 on the mRNA Expression of Key Host Genes Involved in Glutamine Metabolism During WSSV Infection

Following the protocol described in previous studies (8, 9, 19), shrimp were intramuscularly injected with 100 μl of the inhibitors Salirasib, Torin1, and LY294002 or their vehicles 2 h before being challenged with WSSV. Samples were then collected at 12 and 24 h post WSSV injection. To evaluate the involvement of shrimp Ras, shrimp were treated with Salirasib (dissolved in 99% EtOH and diluted with PBS, pH 8.0; 35 μg/g shrimp) or with vehicle only (0.3% ethanol). Samples from Salirasib-pretreated shrimp were collected into 4 pooled hemocyte samples, with each sample being taken from 3 shrimp (19). To investigate the involvement of the PI3K-mTORC1 pathway, shrimp were treated with LY294002 (dissolved in 10% DMSO and diluted with PBS; 0.625 μg/g shrimp) or with vehicle only (0.01% DMSO). Hemocyte samples from LY294002-pretreated shrimp were collected individually for each group (8). To observe the involvement of mTORC1/mTORC2, shrimp were treated with Torin 1 (dissolved in DMSO and diluted with PEG solvent; 20 μg/g shrimp) or with vehicle only (0.25% PEG, 0.25% Tween 20, and 0.15 M NaCl) five or six pleopod samples were collected from each group, with each sample being taken from 10 shrimp (9). To determine the mRNA expression levels of the target genes, all samples were analyzed by real-time PCR as described above.

## Results

### *In vivo* Tracking of [U-^13^C] Glutamine-Derived Metabolites Showed That Both Glutaminolysis and Reductive Carboxylation Were Activated at the WSSV Genome Replication Stage (12 hpi)

To investigate the effect of WSSV in WSSV-infected hemocytes, shrimp were challenged with WSSV, and then injected with [U-^13^C]glutamine (M5 gln) to allow tracing of this stable carbon isotope through the glutamine metabolic pathways as shown in Figure 1A.

**Figure 2:**
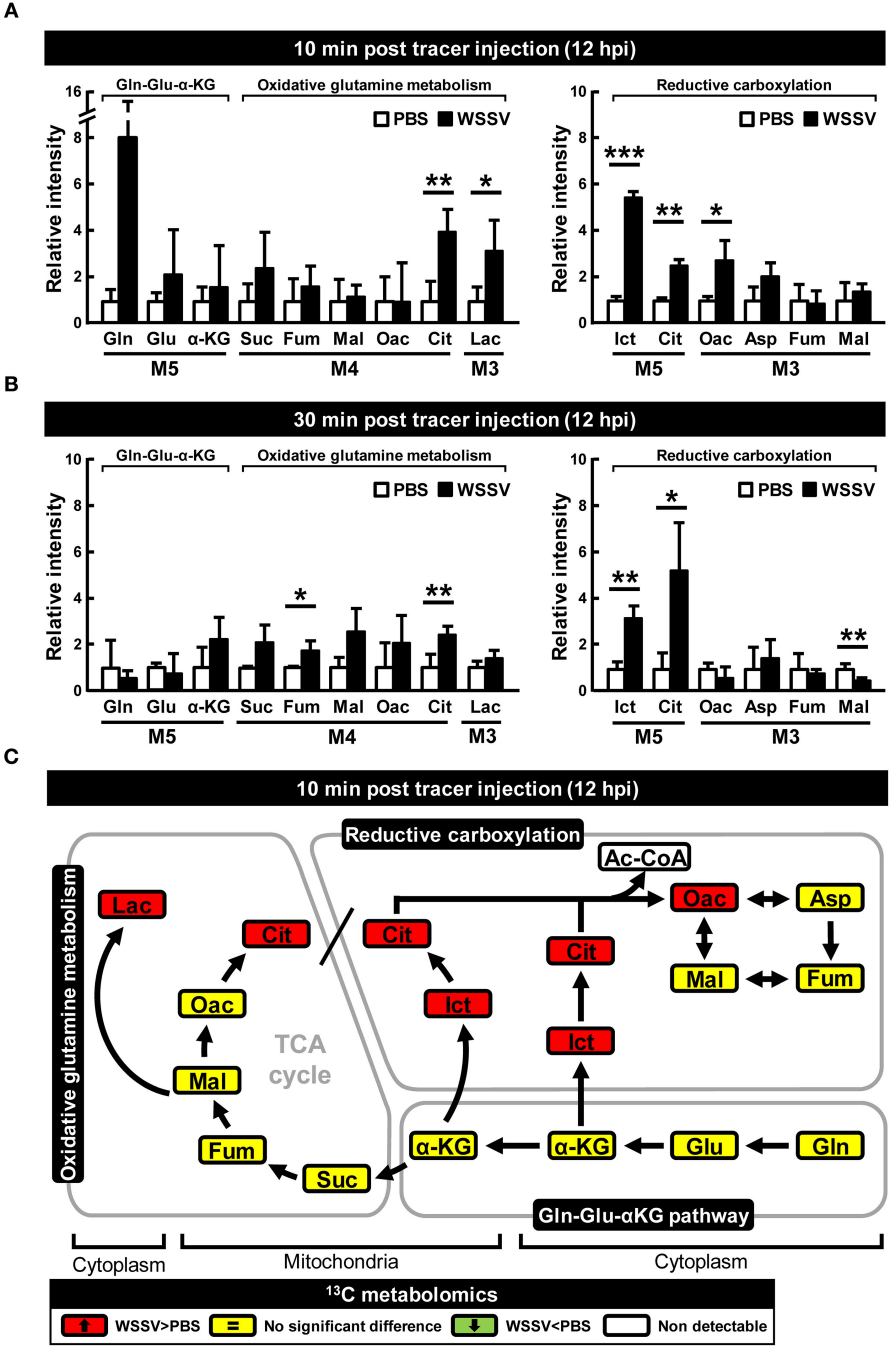
WSSV increased ^13^C-labeled metabolites in oxidative glutaminolysis and reductive carboxylation at the WSSV replication stage (12 hpi). At 12 h after challenge with WSSV or PBS, shrimps were injected with [U-^13^C]glutamine and hemocytes were collected **(A)** 10 min or **(B)** 30 min later. Metabolomic analysis of pooled hemocyte samples by LC-ESI-Q-TOF-MS was used to calculate the fold change of each ^13^C metabolite in the WSSV group compared to the corresponding ^13^C metabolite in the PBS group. Each bar represents the mean ± SD. Asterisks indicate statistically significant differences between the WSSV group and the corresponding PBS control group (*p < 0.05, **p < 0.01, ***p < 0.001). **(C)** Summary of changes in ^13^C glutamine metabolites in the oxidative and reductive directions at 12 hpi and 10 min after [U-^13^C]glutamine treatment. Changes in the WSSV group relative to the corresponding PBS control are color coded as follows: green (significant decrease), yellow (no significant difference), red (significant increase), and white (non-detectable). Abbreviations are the same as Figure 1.

Compared to the PBS treated shrimp, at 12 hpi, there was an 8-fold increase in the amount of M5 glutamine in shrimp hemocytes at 10 min after treatment with [U-^13^C]glutamine (Figure 2A). At the same point, there was also a significant increase in the final downstream product of oxidative glutaminolysis, M3 lactate (Figure 2A). These data suggest that, at 12 hpi, WSSV infection triggers glutamine uptake into shrimp hemocytes and that at least some of this glutamine is converted into lactate via oxidative glutaminolysis. Figure 2A also shows a significant increase in M5 isocitrate and M5 citrate which suggests that the glutamine-dependent reductive carboxylation pathway is also active in WSSV-infected shrimp hemocytes at 12 hpi. At 30 min after [U-^13^C]glutamine treatment, similarity elevated amounts of the oxidative and reductive pathway metabolites were still detected (Figure 2B), but the relatively low levels of glutamine and downstream products such as M4 citrate and M3 oxaloacetate suggest that most of the labeled glutamine input might already have flushed through these pathways. Figure 2C summarizes the changes in these ^13^C glutamine metabolites in both the oxidative and reductive directions at 12 hpi and 10 min after [U-^13^C]glutamine treatment. The raw metabolomic data for [U-^13^C]glutamine and its metabolites are given in Table S1.

### *In vivo* Tracking of [U-^13^C] Glutamine-Derived Metabolites Suggests That Glutaminolysis and Reductive Carboxylation are No Longer Activated at the Late Stage of the WSSV Replication Cycle (24 hpi)

**Figure 3:**
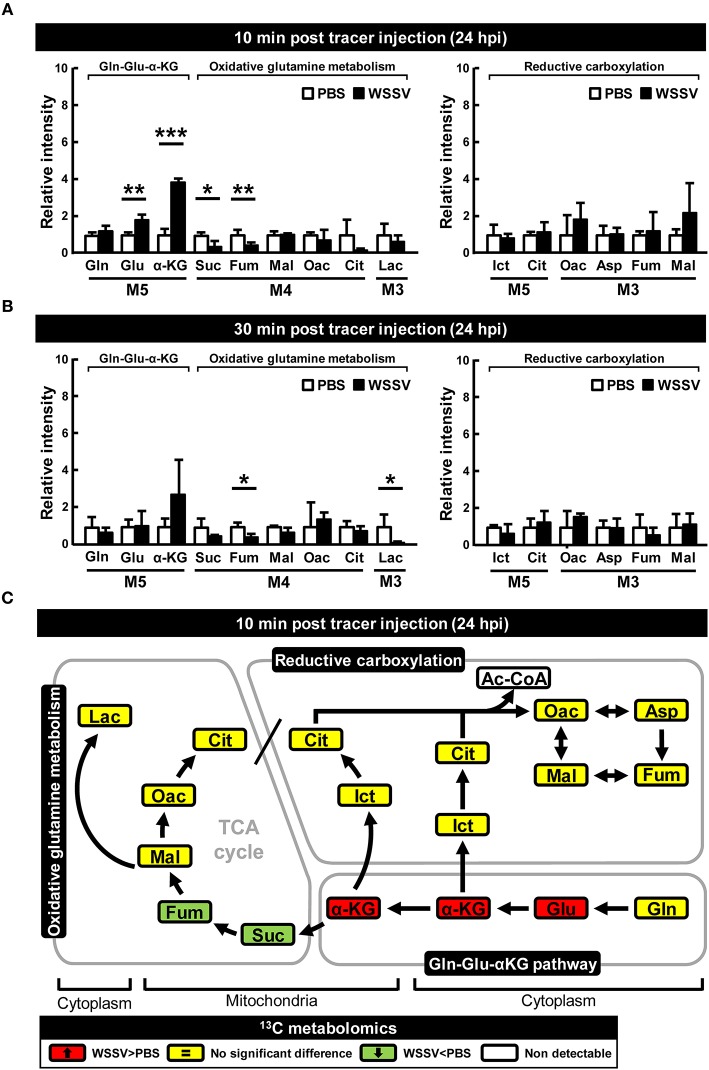
WSSV suppressed oxidative glutaminolysis and reductive carboxylation at the late stage of WSSV replication (24 hpi). At 24 h after challenge with WSSV or PBS, shrimps were injected with [U-^13^C]glutamine and hemocytes were collected **(A)** 10 min or **(B)** 30 min later. Metabolomic analysis of pooled hemocyte samples by LC-ESI-Q-TOF-MS was used to calculate the fold change of each ^13^C metabolite in the WSSV group compared to the corresponding ^13^C metabolite in the PBS group. Each bar represents the mean ± SD. Asterisks indicate statistically significant differences between WSSV group and the corresponding PBS control group (*p < 0.05, **p < 0.01, ***p < 0.001). **(C)** Schematic representation of ^13^C metabolic expression of glutamine metabolism in oxidative and reductive directions at 24 hpi at 10 min after [U-^13^C]glutamine treatment. Changes in the WSSV group relative to the corresponding PBS control are color coded as follows: green (significant decrease), yellow (no significant difference), red (significant increase), and white (non-detectable). Abbreviations are the same as for Figure 1.

At 24 hpi, although [U-^13^C]glutamine still seems to be taken up by the WSSV-infected hemocytes, the elevated accumulation of M5 α-KG and the reduced levels of the glutamine-derived TCA intermediates (i.e., the M4 forms of succinate, fumarate, malate, oxaloacetate, and the M3 forms of lactate) suggest that oxidative metabolism of [U-^13^C]glutamine was not triggered at this stage (Figures 3A,B). Similarly, there was no evidence of reductive carboxylation, i.e., no obvious increase in the M5 forms of isocitrate and citrate or the M3 forms of oxaloacetate, aspartate, fumarate, and malate (Figures 3A,B). Figure 3C provides a graphical summary of the above results.

### *In vivo* Tracking of [1-^13^C] Glutamine-Derived Metabolites Reconfirmed That Reductive Carboxylation Was Activated at the WSSV Genome Replication Stage (12 hpi)

**Figure 4:**
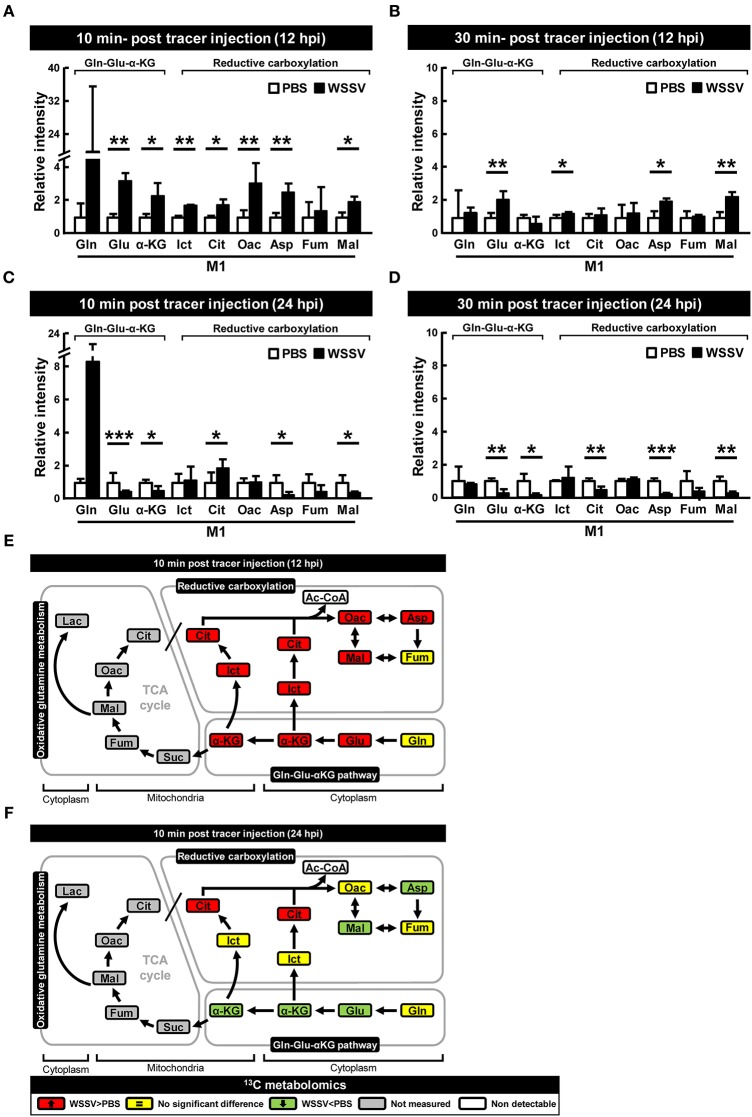
WSSV activates reductive carboxylation at the WSSV genome replication stage (12 hpi) and suppresses it at the late stage of WSSV replication (24 hpi). At 12 h after challenge with WSSV or PBS, shrimps were injected with [1-^13^C]glutamine and hemocytes were collected **(A)** 10 min or **(B)** 30 min later. At 24 h after challenge with WSSV or PBS, shrimps were also injected with [1-^13^C]glutamine and hemocytes were collected **(C)** 10 min or **(D)** 30 min later. Metabolomic analysis of pooled hemocyte samples by LC-ESI-Q-TOF-MS was used to calculate the fold change of each ^13^C metabolite in the WSSV group compared to the corresponding ^13^C metabolite in the PBS group. Each bar represents the mean ± SD. Asterisks indicate statistically significant differences between WSSV group and the corresponding PBS control group (*p < 0.05, **p < 0.01, ***p < 0.001). **(E,F)** Schematic representation of ^13^C metabolic expression of glutamine metabolism in the reductive direction at **(E)** 12 and **(F)** 24 hpi at 10 min after [1-^13^C]glutamine treatment. Changes in the WSSV groups relative to the corresponding PBS controls are color coded as follows: green (significant decrease), yellow (no significant difference), red (significant increase), gray (not measured), and white (non-detectable). Abbreviations are the same as for Figure 1.

The above isotope-labeled tracing experiments were repeated using [1-^13^C]glutamine instead of [U-^13^C]glutamine. With [1-^13^C]glutamine, the first isotope-labeled carbon is lost during oxidative conversion from α-KG to succinate, while this same labeled carbon is kept in the metabolites produced by reductive carboxylation (Figure 1B). Compared to the PBS-treated shrimp, there was generally an increase of M1 metabolites in the glutamine-glutamate-αKG (Gln-Glu-α-KG) pathway in shrimp hemocytes at the WSSV genome replication stage (12 hpi; Figures 4A,B), and there was also a general increase in the amounts of the subsequent metabolites in the reductive carboxylation pathway (Figures 4A,B). As before, these results suggest that reductive carboxylation is triggered at the WSSV genome replication stage. However, once again, there was no clear pattern of increase in these metabolites at the late stage of WSSV replication (Figures 4C,D). Figures 4E,F provide graphical summaries of these results. Raw metabolomic data is given in Table S2.

### WSSV Activates Glutamine Metabolism-Related Genes in *L. vannamei* at the Genome Replication Stage

**Figure 5:**
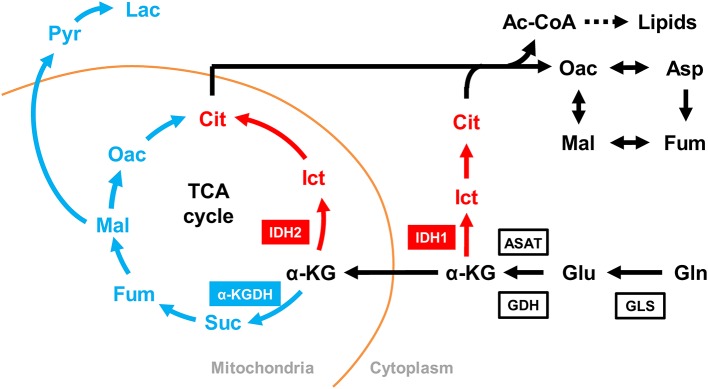
Simplified schematic of oxidative and reductive glutamine metabolic pathways. Glutamine metabolism in the oxidative direction (glutaminolysis) is shown in blue, glutamine metabolism in the reductive direction (reductive carboxylation) is shown in red, and shared metabolic pathways are shown in black. Enzymes: GLS, glutaminase; GDH, glutamine dehydrogenase; ASAT, aspartate aminotransferase; α-KG, α-ketoglutarate; IDH, isocitrate dehydrogenase; α-KGDH, α-ketoglutarate dehydrogenase. Metabolites: abbreviations are the same as for Figure 1.

Previous reports have shown that at the WSSV genome replication stage (12 hpi), WSSV activates GDH and ASAT to induce glutamine metabolism, which in turn fuels the TCA cycle via α-KG (8). Here, to further investigate the enzymes involved in WSSV-induced glutamine metabolism, we next isolated and characterized 5 other important genes involved in this pathway, *Lv*GLS1, *Lv*GLS2, *Lv*IDH1, *Lv*IDH2, and *Lv*α-KGDH (Figure 5). The full-length or partial cDNAs of these 5 genes were identified from our in-house transcriptomic database, and after amplification by PCR with the corresponding primer set (Table 1), the amplicons were sequenced and confirmed to match their respective genes (data not shown).

**Figure 6:**
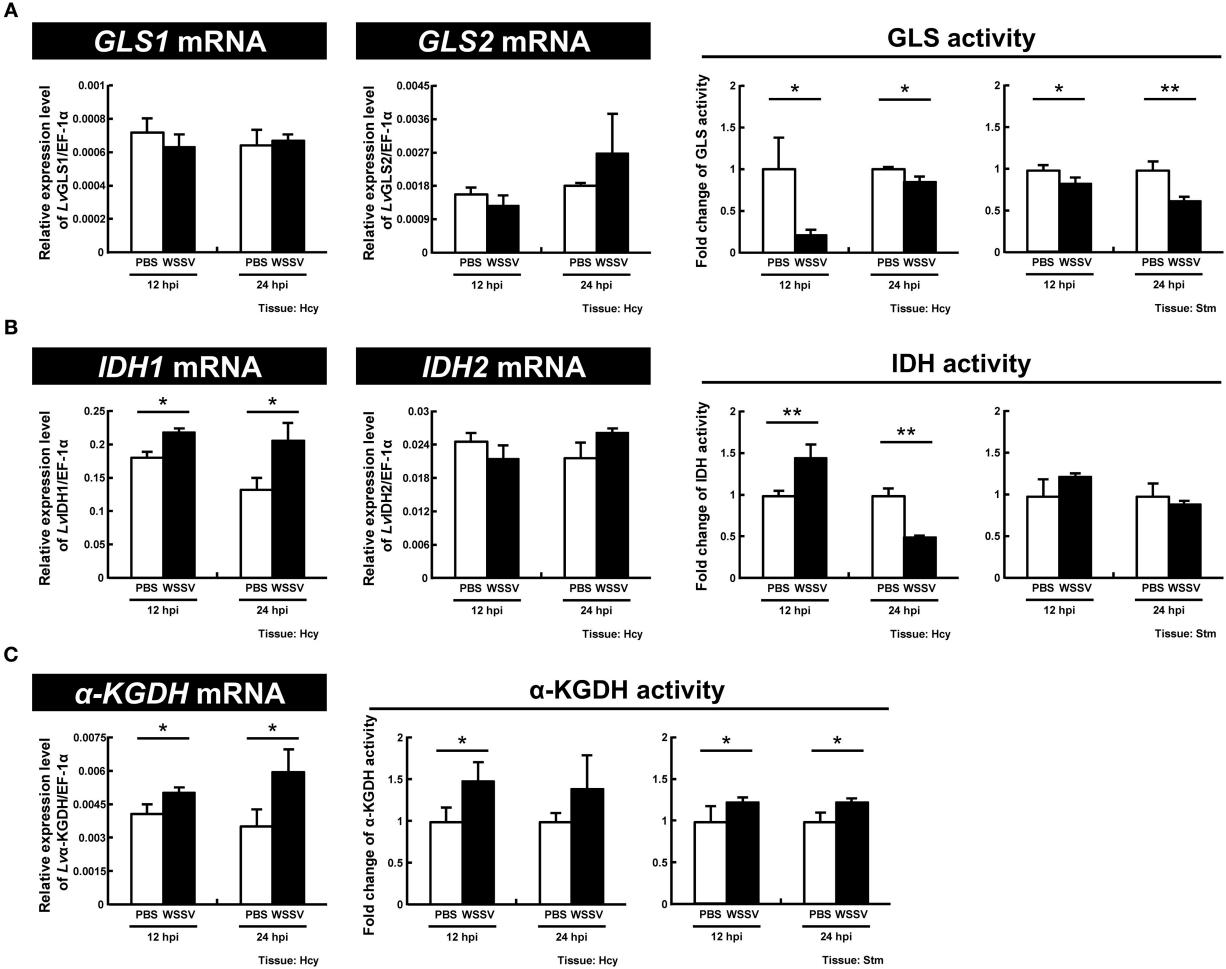
WSSV induces IDH and α-KGDH expression at the WSSV replication stage (12 hpi) in shrimp hemocytes. **(A)** The mRNA levels of GLS1 and GLS2 and the GLS enzyme activity in shrimp hemocytes and stomachs during WSSV infection. **(B)** The mRNA levels of IDH1 and IDH2 and the IDH enzyme activity in shrimp hemocytes and stomachs during WSSV infection. **(C)** The mRNA level and the enzyme activity of α-KGDH in shrimp hemocytes and stomachs during WSSV infection. Each bar represents the mean ± SD. Asterisks indicate statistically significant differences between the WSSV group and the corresponding PBS control group (*p < 0.05, **p < 0.01).

For GLS, which functions as the initial enzyme in glutamine metabolism by converting glutamine to glutamate, we found that although there was no significant change in the expression of the two GLS isoforms, GLS1 and GLS2, the enzyme activity was significantly decreased at both stages of the WSSV replication cycle (12 and 24 hpi) in both hemocytes and stomach (Figure 6A). For IDH1 and IDH2, which are the key enzymes in reductive glutamine metabolism, significant upregulation of IDH1 mRNA expression was observed at 12 and 24 hpi, while there was no change in IDH2. There was also a significant increase in the enzyme activity of IDH in shrimp hemocytes at 12 hpi, and a significant decrease at 24 hpi; meanwhile in the stomach there was no change (Figure 6B). For α-KGDH, which is a key enzyme in oxidative glutamine metabolism, the mRNA levels and enzyme activity were significantly increased in hemocytes at both 12 and 24 hpi (Figure 6C). α-KGDH enzyme activity was also significantly increased in shrimp stomach (Figure 6C). Taken together, at least at 12 hpi, WSSV seems to trigger both IDH1-mediated reductive glutamine metabolism as well as α-KGDH-mediated glutamine metabolism.

### Genes Involved in Oxidative Glutaminolysis and Reductive Glutamine Metabolism Are Important for WSSV Replication

**Figure 7:**
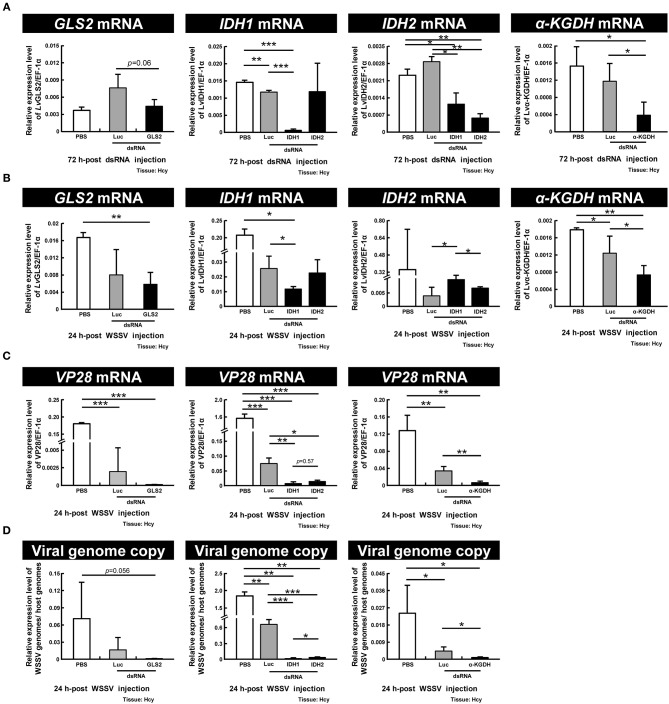
IDH1, IDH2, α-KGDH, and to a lesser extent, GLS are involved in WSSV replication. **(A)** Gene expression of GLS2, IDH1, IDH2, and α-KGDH in shrimp hemocytes was analyzed by real-time PCR at 72 h-post injection of the corresponding dsRNA and before WSSV challenge. **(B)** Gene expression of the above genes was measured again in dsRNA-treated shrimp at 24 h post WSSV injection. **(C,D)** The effect of gene silencing of GLS2, IDH1, IDH2, and α-KGDH on the expression of the WSSV gene VP28 and WSSV genome copy numbers at 24 h post WSSV injection. Groups treated with PBS only or with non-specific luciferase (Luc) dsRNA were used as control groups. Each bar represents the mean ± SD. Asterisks indicate statistically significant differences between the indicated groups (*p < 0.05, **p < 0.01, ***p < 0.001).

To further investigate the importance of GLS2, IDH1, IDH2, and α-KGDH in WSSV replication, we performed dsRNA-mediated *in vivo* silencing of these genes with the corresponding dsRNAs. (Unfortunately, GLS1 dsRNA could not be synthesized because the sequence of this gene has not yet been completely determined, and we failed to find any primer set that could successfully produce any PCR amplicons.) As Figure 7A shows, at 72 h-post dsRNA treatment, although the gene expression of GLS2 was unchanged relative to PBS control, the gene expressions of IDH1, IDH2, and α-KGDH were all specifically decreased. After this time point, the shrimp were then injected with WSSV and hemocytes were collected 24 h later. We found that although the mRNA expression of IDH1 and α-KGDH were still significantly suppressed by the corresponding dsRNAs and that GLS2 was also suppressed at this time, the mRNA level of IDH2 was not significantly different from the PBS or Luciferase controls (Figure 7B). We further found that there was significant suppression of WSSV VP28 mRNA expression and WSSV viral copy numbers in the WSSV-injected groups pretreated with IDH1, IDH2, and α-KGDH dsRNA, while in the GLS2 group, VP28 mRNA was significantly suppressed even though the viral copy number was not (Figures 7C,D). Taken together, these data suggest that IDH1, IDH2, α-KGDH, and to a lesser extent, GLS might all be important for WSSV replication.

### Ras Is a Primary Regulator of Reductive Glutamine Metabolism at the WSSV Genome Replication Stage

**Figure 8:**
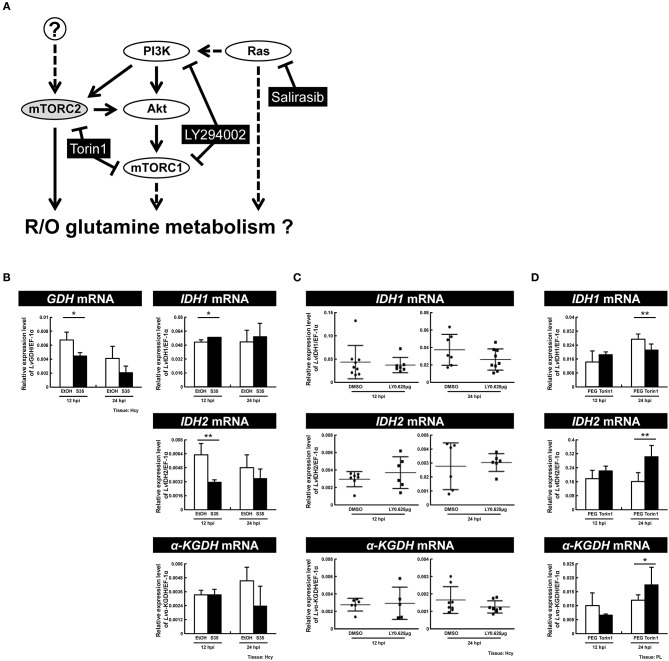
Ras is a primary regulator of reductive glutamine metabolism at the WSSV genome replication stage. **(A)** Schematic representation of the Ras and PI3K-Akt-mTOR pathways and the three inhibitors used in this experiment (black boxes). Shrimp were treated with the various inhibitors or vehicle only 2 h before WSSV infection. **(B)** Real-time PCR analysis shows that the RAS inhibitor Salirasib had a significant effect on the expression of GDH, IDH1, and IDH2 in shrimp hemocytes after WSSV infection. **(C)** Real-time PCR analysis shows that the PI3K-mTOR inhibitor LY29400 had no significant effect on the expression of IDH1, IDH2, and α-KGDH in shrimp hemocytes after WSSV infection. **(D)** Real-time PCR analysis shows that the mTORC1/C2 inhibitor Torin 1 had a significant effect on the expression of IDH1, IDH2, and α-KGDH in shrimp pleopods after WSSV infection. Bars represent the mean ± SD. Asterisks indicate statistically significant differences in WSSV-injected shrimp between the inhibitor treated groups and the corresponding vehicle-only control (*p < 0.05, **p < 0.01).

Previous reports have suggested that the PI3K-Akt-mTOR pathway is involved in triggering both the WSSV-induced Warburg effect at 12 hpi (9) and WSSV-induced lipogenesis at 24 hpi (7). Meanwhile, mTORC2 is a key regulator for upregulating the expression of GDH mRNA independently of PI3K-mTORC1 at 12 hpi (8), while LvRas has been shown to play a positive role in triggering WSSV-induced PI3K-Akt-mTOR activation (19). To investigate which of these pathways might regulate oxidative and reductive glutamine metabolism during WSSV infection, shrimp were treated with three specific inhibitors (Figure 8A) and the mRNA expression of key genes involved in glutamine metabolism was monitored.

In shrimps treated with Salirasib, a Ras inhibitor which disrupts the spatiotemporal localization of active Ras (21), the gene expression of GDH and IDH2 was significantly decreased at 12 hpi (Figure 8B). At the same time, IDH1 mRNA was significantly increased. At 24 hpi, inhibition of Ras caused no change in the mRNA levels of these glutamine metabolism-related genes. These data suggest that Ras not only upregulates the mRNA level of GDH, but also drives an increase in reductive glutamine metabolism by increasing the mRNA levels of IDH2 at the WSSV genome replication stage.

To investigate the role of the PI3K-Akt-mTOR pathway, shrimps were pretreated with LY294002 to inhibit both PI3K and mTORC1. In LY294002-treated shrimps, at both 12 and 24 hpi, there was no significant difference in the mRNA levels of IDH1, IDH2, or α-KGDH compared to the control group (Figure 8C). In shrimps treated with Torin1, which was used to inhibit both of the mTOR complexes, there was no impact on any of the three enzymes at 12 hpi, whereas, at 24 hpi, gene expression of IDH1 decreased while IDH2 and α-KGDH mRNA increased (Figure 8D). Taken together, these results suggest that at 12 hpi, glutamine metabolism is regulated by Ras but not by the PI3K-Akt-mTOR pathway. Meanwhile, at 24 hpi, mTORC2 is pre-dominantly responsible for the down-regulation of glutamine metabolism, and it acts independently of Ras and the PI3K-mTORC1 pathway.

## Discussion

We have shown here that, as in other cancer or virally infected cells that are in a state of aerobic glycolysis (10, 22, 23), both oxidative glutaminolysis and reductive carboxylation have been triggered in WSSV-infected shrimp hemocytes (Figures 2–4). The present results are mostly consistent with previous findings for WSSV-induced glutaminolysis. For example, the significantly increased levels of M4 citrate in Figure 2A can be accounted for by the upregulation of citrate synthase at the protein level, as reported by Su et al. (9). Further, the patterns of changes in the levels of various metabolites suggest that these pathways might be more active at the genome replication stage (12 hpi) than in the late stage (24 hpi). This conclusion is further supported by our dsRNA silencing results for several key enzymes. In particular, we found that in *L. vannamei* hemocytes, IDH activity was induced at 12 hpi, although only cytoplasmic IDH1 showed an increase in mRNA expression, while the mitochondrial IDH2 was unchanged (Figure 6). Shrimp IDH1 silencing also led to stronger suppression of the WSSV genome copy number (Figure 7). From this we infer that cytoplasmic IDH1 is likely to play a relatively more important role in triggering WSSV-induced reductive carboxylation. We also note that IDH1 plays a key role in NADPH production and acts as a potential regulator of lipid metabolism in cancer cells and in virus-infected cells with reductive metabolism (23, 24). At the same time (12 hpi), there was also an increase in α-KGDH activity (Figure 6B), suggesting that the TCA cycle was also upregulated in the oxidative direction. Again, the importance of this enzyme to WSSV replication was shown by the significant reduction in VP28 mRNA and viral genome copies when α-KGDH expression was silenced by dsRNA (Figures 7C,D). We note too that since enzymes such as IDH1 and α-KGDH appear to be necessary for the virus to replicate, they might also be useful as biomarkers for developing disease-resistant shrimp.

While oxidative glutamine metabolism provides an alternative mechanism to produce ATP and, like glycolysis, results in the accumulation of lactate as an end product, the reductive carboxylation pathway is important for providing the macromolecular precursors of lipid synthase (18, 23, 24). In a previous study, we found that lipogenesis, which is essential for the creation of the lipid-containing fraction of the WSSV viral envelope, was induced at 24 hpi (7). Here, however, our results suggest that reductive carboxylation might be more active at 12 hpi rather than 24 hpi (Figures 2–4), which would imply that although lipogenesis might still occur at the late stage, the lipid macromolecular precursors are in fact being produced at an earlier time point than was previously proposed. Alternatively, following Munger et al. (25), who showed that HCMV was able to divert glucose-derived carbon away from lactate and into fatty acid synthesis, it is also possible that in the same way, the glycolysis pathway might act as the carbon source for lipid synthesis in WSSV-infected cells that are in a state of aerobic glycolysis. Unfortunately, in the present study, we were unable to resolve this question because the Ac-CoA metabolite could not be detected, but we are currently working on a new set of experiments that use ^13^C-labeled glucose to trace Ac-CoA's carbon source.

We found in an earlier study that although WSSV stimulates glutamine metabolism to fuel the TCA cycle via α-KG (8), the carbon source that was taken up by the WSSV-infected hemocytes was glutamate rather than glutamine. In the present study, our stable isotope results (Figures 2–4) clearly show that glutamine is also being taken up and metabolized by the WSSV-infected hemocytes. The first step in this metabolic pathway is the conversion of glutamine to glutamate by GLS, and it is interesting to note that while WSSV upregulates the activity of both GDH and ASAT (8), we found here that the mRNA expression levels of shrimp GLS were unchanged and its enzyme activity was actually reduced (Figure 6A). This is also in contrast to other similar instances of metabolic reprogramming during aerobic glycolysis, such as HCMV for example, where glutaminolysis is associated with the increased activity of both GLS and GDH (26). In the case of WSSV, we therefore hypothesize that the increased uptake of glutamine might instead be driven by the high demand created by the conversion of glutamate to α-KG. Further, although our experiments here demonstrate that labeled glutamine can be used as the carbon source, as noted above, our previous study found that WSSV-induced glutaminolysis was driven by glutamate rather than glutamine (8). This is consistent with the observed down-regulation of GLS activity (Figure 6A), because although this enzyme would still provide some of the carbon input, it would no longer be essential for driving this metabolic pathway.

**Figure 9:**
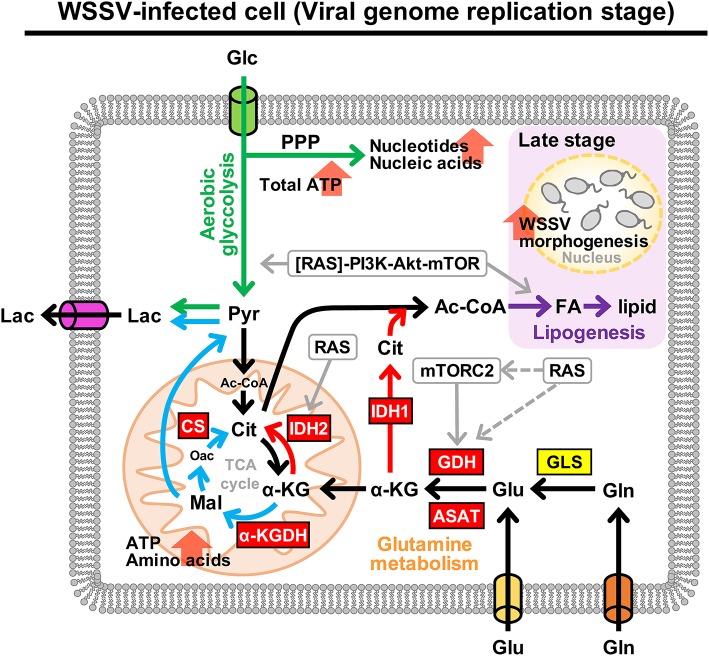
Schematic representation of metabolism in WSSV-infected hemocytes showing the aerobic glycolysis, glutamine metabolism and lipogenesis pathways at the WSSV genome replication stage (12 hpi). This figure was compiled from data obtained in the present study and previous studies (7–9, 19). Enzymes are shown in boxes where red indicates an upregulated enzyme and yellow indicates the enzyme was unchanged. Oxidative glutamine metabolism and reductive carboxylation are indicated by blue and red arrows, respectively. Green arrows represent aerobic glycolysis and the pentose phosphate pathway (PPP). WSSV-triggered lipogenesis, which is triggered at the late stage, is shown in purple. Round boxes and gray arrows indicate enzyme activity and signaling pathways. Pyr, pyruvate; CS, citrate synthase; FA, fatty acids. All other abbreviations are the same as for Figure 1.

Figure 9 provides a comprehensive schematic that summarizes the increased mRNA levels of all of the above enzymes in shrimp hemocytes at 12 hpi when the Warburg effect is activated. The same figure also shows the larger context, including the PI3K-Akt-mTOR pathway [by which the Warburg effect is triggered; (9)] and the means by which WSSV is able to replenish the TCA cycle by triggering glutamine metabolism, i.e., the Gln-Glu-α-KG pathway, oxidative glutamine metabolism and reductive carboxylation.

In our previous research, we found that WSSV induces glutamine metabolism independently of the PI3K-Akt-mTORC1 pathway and instead uses mTORC2 to up-regulate GDH mRNA (8). Here we further showed that although Ras can sometimes act as an upstream regulator of the PI3K-Akt-mTOR and Raf-MEK-ERK pathways (27, 28), in WSSV-infected hemocytes, it acts independently to significantly upregulate the gene expression of GDH (Figure 8B). Taken together, it thus appears that Ras and mTORC2 may both regulate the gene expression level of GDH. As shown in Figure 9, however, their exact relationship remains unclear. Although it is possible that Ras acts directly as a GDH regulator, in *Dictyostelium* it promotes cell migration via the activation of mTORC2 (29). mTORC2 has also been reported to encode a Ras binding domain that may be correlated to Ras regulation (30), and in cancer research, oncogenic Ras binds to mTORC2 and stimulates its activation to promote cell proliferation and tumorigenesis (31). Further research will be needed to establish whether or not the effect of Ras on GDH is mediated by mTORC2 in a similar way. Lastly, there remaining the question of which viral factors might be involved in this metabolic reprogramming. In the case of dengue virus, which leads to increased glycolysis in the host, Allonso et al. found that viral NS1 protein interacts with glyceraldehyde-3-phosphate dehydrogenase (GAPDH) and upregulates its activity (32). To address the question of how WSSV might regulate glutamine metabolism, we are now working with a yeast two-hybridization platform that has so far been used to select several viral factor candidates that can interact with enzymes related to this pathway. We hope to further explore these potential mechanisms in a future study.

## Data Availability

All datasets generated for this study are included in the manuscript/Supplementary Files.

## Author Contributions

S-TH, C-YT, and C-YL designed and performed *in vivo* animal experiments and analyzed data. D-YL performed LC-ESI-MS-based isotopic labeled metabolomic analysis. S-TH, D-YL, and H-CW wrote the manuscript. H-CW conceived the idea, designed the research, discussed data, and supervised this work.

### Conflict of Interest Statement

The authors declare that the research was conducted in the absence of any commercial or financial relationships that could be construed as a potential conflict of interest.

## Abbreviations

WSSV: white spot syndrome virus
LC-ESI-MS: Liquid chromatography-electrospray ionization mass spectrometry
PBS: phosphate-buffered saline
GLS: glutaminase
GDH: glutamine dehydrogenase
ASAT: aspartate aminotransferase
IDH: isocitrate dehydrogenase
α-KGDH: α-ketoglutarate dehydrogenase.
Gln: glutamine
Glu: glutamate
α-KG: α-ketoglutarate
Suc: succinate
Fum: fumarate
Mal: malate
Oac: oxaloacetate
Cit: citrate
Ict: isocitrate
Lac: lactate
Asp: aspartate.
