# Supplementary material for: Glutamine Metabolism in Both the Oxidative and Reductive Directions Is Triggered in Shrimp Immune Cells (Hemocytes) at the WSSV Genome Replication Stage to Benefit Virus Replication
Source: Front Immunol. 2019 Sep 4;10:2102. doi: 10.3389/fimmu.2019.02102 (PMC6737011; doi:10.3389/fimmu.2019.02102)
Supplement: Supplementary file 1 [file Table_1.DOCX]

Supplementary Material

# Supplementary Tables

## Table S1. Quantitative changes in the oxidative glutaminolysis and reductive carboxylation metabolites in WSSV-infected shrimp hemocytes as determined by [U-^13^C]glutamine (M5 Gln) tracing.

| **Metabolites** | | | **10 min** | | | **30 min** | | |
| --- | --- | --- | --- | --- | --- | --- | --- | --- |
|  |  |  | **PBS** | **WSSV^a^** | **Fold**  **change^b^** | **PBS** | **WSSV^a^** | **Fold change^b^** |
| **Oxidative glutaminolysis** | **12 hpi** | **M5 Gln** | 45.1±20.5 | 359.4±333.3 | 8.0 | 135.3±163.6 | 85.4±33.0 | 0.6 |
|  |  | **M5 Glu** | 90.0±28.4 | 197.3±169.9 | 2.2 | 102.3±147.5 | 258.6±259.0 | 2.5 |
|  |  | **M5 α-KG** | 156.5±86.7 | 254.9±279.5 | 1.6 | 193.0±173.9 | 436.9±174.0 | 2.3 |
|  |  | **M4 Suc** | 1091.0±778.6 | 2671.2±1610.9 | 2.4 | 2492.8±4.0 | 5368.4±1739.0 | 2.2 |
|  |  | **M4 Fum** | 48.5±45.3 | 79.6±40.5 | 1.6 | 717.0±7.4 | 1295.8±264.1 | 1.8 |
|  |  | **M4 Mal** | 314.784±305.4 | 389.7±136.8 | 1.2 | 1061.7±453.4 | 2798.9±990.5 | 2.6 |
|  |  | **M4 Oac** | 413.887±422.5 | 406.0±677.3 | 1.0 | 166.0±181.5 | 354.6±191.2 | 2.1 |
|  |  | **M4 Cit** | 451.1±363.8 | 1813.5±384.1 | 4.0 | 1190.0±643.1 | 2952.9±385.6 | 2.5 |
|  |  | **M3 Lac** | 59.9±33.5 | 190.4±76.5 | 3.2 | 511.5±123.1 | 735.5±150.6 | 1.4 |
|  | **24 hpi** | **M5 Gln** | 211.3±20.4 | 224.0±82.1 | 1.1 | 274.7±125.8 | 193.0±58.6 | 0.7 |
|  |  | **M5 Glu** | 2428.0±303.0 | 3798.3±415.4 | 1.6 | 1815.7±660.8 | 1901.5±1356.0 | 1.0 |
|  |  | **M5 α-KG** | 583.4±198.9 | 2258.8±81.8 | 3.9 | 400.7±158.4 | 1103.8±718.0 | 2.8 |
|  |  | **M4 Suc** | 9356.9±950.2 | 3852.2±1713.0 | 0.4 | 23523.6±9139.7 | 11982.1±591.0 | 0.5 |
|  |  | **M4 Fum** | 1012.0±235.8 | 404.0±129.0 | 0.4 | 4244.7±768.9 | 1864.9±584.9 | 0.4 |
|  |  | **M4 Mal** | 3216.8±600.8 | 3270.8±55.6 | 1.0 | 11505.0±466.3 | 7958.4±3015.9 | 0.7 |
|  |  | **M4 Oac** | 291.6±32.7 | 200.6±165.3 | 0.7 | 546.5±706.7 | 752.7±188.3 | 1.4 |
|  |  | **M4 Cit** | 5834.3±4568.5 | 596.0±761.5 | 0.1 | 11948.3±3281.1 | 9750.3±2437.6 | 0.8 |
|  |  | **M3 Lac** | 192.1±101.1 | 108.5±68.0 | 0.6 | 1812.1±1084.7 | 203.6±118.2 | 0.1 |
| **Reductive carboxylation** | **12 hpi** | **M5 Ict** | 119.8**±**15.3 | 652.2**±**24.3 | 5.4 | 278.4±81.1 | 904.3±130.0 | 3.2 |
|  |  | **M5 Cit** | 604.8**±**61.3 | 1523.8**±**132.0 | 2.5 | 287.4±186.2 | 1511.6±590.6 | 5.3 |
|  |  | **M3 Oac** | 37.8**±**6.1 | 103.1**±**32.0 | 2.7 | 310.0±61.2 | 193.6±132.9 | 0.6 |
|  |  | **M3 Asp** | 709.7**±**369.7 | 1429.2**±**411.4 | 2.0 | 2325.5±2098.9 | 3405.1±1834.0 | 1.5 |
|  |  | **M3 Fum** | 248.6**±**173.6 | 214.2**±**129.1 | 0.9 | 385.4±244.9 | 301.8±47.5 | 0.8 |
|  |  | **M3 Mal** | 173.2**±**131.4 | 239.8**±**51.9 | 1.4 | 229.9±36.9 | 105.6±22.2 | 0.5 |
|  | **24 hpi** | **M5 Ict** | 2918.0±1436.0 | 2409.6±664.2 | 0.8 | 3198.2±149.0 | 2132.5±1458.8 | 0.7 |
|  |  | **M5 Cit** | 2855.3±296.9 | 3341.8±1348.1 | 1.2 | 2948.2±1313.3 | 3651.6±1731.7 | 1.2 |
|  |  | **M3 Oac** | 203.1±205.9 | 372.5±176.7 | 1.8 | 271.3±226.5 | 423.5±34.9 | 1.6 |
|  |  | **M3 Asp** | 4814.3±2348.3 | 5147.7±1446.5 | 1.1 | 11684.7±3262.5 | 11326.9±5567.1 | 1.0 |
|  |  | **M3 Fum** | 620.0±78.6 | 767.5±586.5 | 1.2 | 1676.0±1097.3 | 920.4±669.3 | 0.5 |
|  |  | **M3 Mal** | 160.2±43.7 | 355.9±251.8 | 2.2 | 275.1±188.3 | 322.0±141.6 | 1.2 |

**^a^:** Red and green indicate significant up- and down-regulation, respectively. Yellow indicates no significant change.

**^b^:** Fold change: WSSV / PBS

## Table S2. Quantitative changes in the reductive carboxylation metabolites in WSSV-infected shrimp hemocytes as determined by [1-^13^C]glutamine (M1 Gln) tracing.

| **Metabolites** | | | **10 min** | | | **30 min** | | |
| --- | --- | --- | --- | --- | --- | --- | --- | --- |
|  |  |  | **PBS** | **WSSV^a^** | **Fold change^b^** | **PBS** | **WSSV^a^** | **Fold change^b^** |
| **Reductive carboxylation** | **12 hpi** | **M1 Gln** | 39.3±29.8 | 793.0±609.4 | 20.2 | 535.2±855.3 | 682.6±154.5 | 1.3 |
|  |  | **M1 Glu** | 1463.1±186.2 | 4677.6±633.9 | 3.2 | 1969.1±487.3 | 4168.9±842.9 | 2.1 |
|  |  | **M1 α-KG** | 3376.7±592.8 | 7879.5±2397.1 | 2.3 | 2765.2±384.5 | 1717.0±1095.8 | 0.6 |
|  |  | **M1 Ict** | 2417.8±149.1 | 4088.4±20.6 | 1.7 | 4107.0±538.9 | 5140.4±203.8 | 1.3 |
|  |  | **M1 Cit** | 7889.6±301.5 | 13948.0±2045.3 | 1.8 | 11367.4±2015.6 | 13613.5±3660.6 | 1.2 |
|  |  | **M1 Oac** | 3166.0±1152.3 | 9694.0±3707.3 | 3.1 | 4334.9±3084.1 | 5558.5±2452.7 | 1.3 |
|  |  | **M1 Asp** | 100798.7±21077.4 | 253490.7±49481.5 | 2.5 | 148638.3±46371 | 298677.8±9177.6 | 2.0 |
|  |  | **M1 Fum** | 3046.1±2785.7 | 4250.7±4349.3 | 1.4 | 6241.4±1962.4 | 6687.1±221.2 | 1.1 |
|  |  | **M1 Mal** | 10406.5±2555.2 | 19811.3±3152.0 | 1.9 | 10185.6±2926.7 | 22998.5±2132.8 | 2.3 |
|  | **24 hpi** | **M1 Gln** | 232.7±39.5 | 1933.3±3281.8 | 8.3 | 241.0±199.9 | 199.4±1.3 | 0.8 |
|  |  | **M1 Glu** | 4540.2±2529.1 | 2191.4±26.6 | 0.5 | 5360.0±730.0 | 1726.1±666.9 | 0.3 |
|  |  | **M1 α-KG** | 7812.2±923.8 | 3581.1±2162.1 | 0.5 | 7249.1±2801.9 | 1319.6±215.3 | 0.2 |
|  |  | **M1 Ict** | 3560.0±1523.0 | 4065.9±2812.0 | 1.1 | 5324.5±79.7 | 6754.8±3125.8 | 1.3 |
|  |  | **M1 Cit** | 13234.7±7564.3 | 25045.3±5281.7 | 1.9 | 27594.4±2904.4 | 13416.7±3974.6 | 0.5 |
|  |  | **M1 Oac** | 6734.6±1692.6 | 6688.4±1786.2 | 1.0 | 5557.5±694.1 | 6149.4±379.1 | 1.1 |
|  |  | **M1 Asp** | 228070.8±95335.9 | 52384.8±27775.8 | 0.2 | 281860.5±30260.2 | 72074.4±3656.0 | 0.3 |
|  |  | **M1 Fum** | 9213.4±4007.2 | 3782.5±3709.7 | 0.4 | 7738.6±4244.9 | 3399.1±1230.4 | 0.4 |
|  |  | **M1 Mal** | 13736.6±5010.8 | 4574.9±1125.3 | 0.3 | 19205.8±4815.6 | 5444.8±266.2 | 0.3 |

**^a^:** Red and green indicate significant up- and down-regulation, respectively. Yellow indicates no significant change.

**^b^:** Fold change: WSSV / PBS
